# Supplementary material for: Formation of secondary allo-bile acids by novel enzymes from gut Firmicutes
Source: Gut Microbes. 2022 Nov 7;14(1):2132903. doi: 10.1080/19490976.2022.2132903 (PMC9645264; doi:10.1080/19490976.2022.2132903)
Supplement: Supplemental Material [file KGMI_A_2132903_SM8834.docx]

**Formation of secondary allo-bile acids**

**by novel enzymes from gut Firmicutes**

Jae Won Lee,^1,2^ Elise S. Cowley,^3,4^ Patricia G. Wolf,^1,2,5,6,a^ Heidi L. Doden,^1,2^ Tsuyoshi Murai,^7^ Kelly Yovani Olivos Caicedo,^8^ Lindsey K. Ly,^1,9^ Furong Sun,^10^ Hajime Takei,^11^ Hiroshi Nittono,^11^ Steven L. Daniel,^12^ Isaac Cann,^1,2,9,13^ H. Rex Gaskins,^1,2,9,14^ Karthik Anantharaman,^3^ João M. P. Alves,^8^ Jason M. Ridlon^1,2,9,14,15,16^

^1^Carl R. Woese Institute for Genomic Biology, University of Illinois Urbana-Champaign, Urbana, IL, USA

^2^Department of Animal Sciences, University of Illinois Urbana-Champaign, Urbana, IL, USA ^3^Department of Bacteriology, University of Wisconsin-Madison, Madison, WI, USA

^4^Microbiology Doctoral Training Program, University of Wisconsin-Madison, Madison, WI, USA ^5^Institute for Health Research and Policy, University of Illinois Chicago, Chicago, IL, USA ^6^University of Illinois Cancer Center, University of Illinois Chicago, Chicago, IL, USA

^7^School of Pharmaceutical Sciences, Health Sciences University of Hokkaido, Hokkaido, Japan. ^8^Department of Parasitology, Institute of Biomedical Sciences, University of São Paulo, São Paulo, Brazil

^9^Division of Nutritional Sciences, University of Illinois Urbana-Champaign, Urbana, IL, USA

^10^Mass Spectrometry Laboratory, School of Chemical Sciences, University of Illinois Urbana- Champaign, IL, USA

^11^Junshin Clinic Bile Acid Institute, Tokyo, Japan

^12^Department of Biological Sciences, Eastern Illinois University, Charleston, IL, USA

^13^Department of Microbiology, University of Illinois Urbana-Champaign, Urbana, IL, USA

^14^Cancer Center at Illinois, Urbana, IL, USA

^15^Center for Advanced Study, University of Illinois Urbana-Champaign, Urbana, IL, USA

^16^Department of Microbiology and Immunology, Virginia Commonwealth University, Richmond, VA, USA

*Corresponding author: Jason M. Ridlon; [jmridlon@illinois.edu](mailto:jmridlon@illinois.edu)

**Table S1.** Strains and plasmids used in this study

| **Strains** | **Relevant description** | **Reference** |
| --- | --- | --- |
| *E. coli*  Top10 | F-, ϕ80d, *lacZ*ΔM15, *end*A1, *rec*A1, *hsd*R17  (r_K_^-^m_K_^-^), *sup*E44, *thi*-1, *gyr*A96, *rel*A1,  Δ(*lac*ZYA-*arg*F) U169 | Invitrogen  (Carlsbad, CA, USA) |
| *E. coli* BL21(DE3) | F-, *ompT*, *hsdSB*(rB− mB−), *gal*, *dcm, rne131* (DE3) | Invitrogen  (Carlsbad, CA, USA) |
| **Plasmids** |  |  |
| pETduet | Two T7 promoters with two MCS, pBR322 replicon, and Amp^R^ | Novagen  (EMD Milipore, CA, USA) |
| pBaiP | Derived from pETDuet, P_T7_-*baiP*-P_T7_-MCS2-T_T7_, and Amp^R^ | This study |
| pBaiJ | Derived from pETDuet, P_T7_-*baiJ*-P_T7_-MCS2-T_T7_, and Amp^R^ | This study |
| pBaiO | Derived from pETDuet, P_T7_-*baiP*-P_T7_-MCS2-T_T7_, and Amp^R^ | This study |
| pBaiA1 | Derived from pETDuet, P_T7_-MCS1-P_T7_-*baiA1*-T_T7_, and Amp^R^ | This study |
| pBaiP-A1 | Derived from pETDuet, P_T7_-*baiP*-P_T7_-*baiA1*-T_T7_, and Amp^R^ | This study |

**Table S2.** Primers used in this study

| **Name** | **Direction** | **Sequence (5’→3’)** |
| --- | --- | --- |
| V1-F | Sense | AGCTTGCGGCCGCATAATGCTTAAGTCGAACAGAAAGTAATCGTA |
| V1-R | Antisense | CGAATTCGGATCCTGGCTGTGGTGATGATGGTGATGGCTGCTGCCCAT |
| V2-F | Sense | TTAACCTAGGCTGCTGCCACCGCTGAGCAATAA |
| V2-R | Antisense | TATATCTCCTTCTTATACTTAACTAATATACTAAGATGGGGAATT |
| BaiP-F | Sense | ATGGGCAGCAGCCATCACCATCATCACCACAGCCAGGATCCGAATTCGATGGCGTCCTACACACCAGGAACCT |
| BaiP-R | Antisense | CTTAAGCATTATGCGGCCGCAAGCTTTACAGCATTTCACGTTGACAAGCCGTGCG |
| BaiJ-F | Sense | ATGGGCAGCAGCCATCACCATCATCACCACAGCCAGGATCCGAATTCGATGGCACATTACGTACCGGGTGCGT |
| BaiJ-R | Antisense | CTTAAGCATTATGCGGCCGCAAGCTTTAAAGCATACTCTGCCCACTAGCG |
| “BaiO”-F | Sense | ATGGGCAGCAGCCATCACCATCATCACCACAGCCAGGATCCGAATTCGATGATCAGCATGATTCGTATCAATC |
| “BaiO”-R | Antisense | CTTAAGCATTATGCGGCCGCAAGCTTTAGAAATTACGCAGCCCTTTGGGC |
| BaiA1-F | Sense | TAGTTAAGTATAAGAAGGAGATATACATATGCATCACCATCATCACCACAGCATGAGTATTATCGTCATCTCGGGATGCGCG |
| BaiA1-R | Antisense | TTATTGCTCAGCGGTGGCAGCAGCCTAGGTTAATCAAAATTGGGTCGGGCGCATAACTGCATC |

**Table S3.** Synthetic DNA sequence used in this study^a^

| **Name** | **Sequence (5’→3’)** |
| --- | --- |
| *baiP*  from *L. scindens* ATCC 35704  (1,692 bp) | ATGGCGTCCTACACACCAGGAACCTATACGGGTAAAGGGTATGGTGTTCGTGGCAAGGTAATCTTGGAAGTCACGTTCAGCGAAGATCGCATTACTGACATCAAGATCGTTAAACATAAGGAGATCTATGGACAGGCCTACGGCCTTGAGAGCTCGCCTTTCGAATACTATATTCCTAAAATCATTGAGCATCAGAGTCTTGCCGTTCCAATGGTTGTGGGCGCGGAGGTTGTTTGCGGCGCTATCGTTCACGCGGTGGCCAGCTGCGTTGAGCAGGCTGGAGGGGATGCGGATGCACTTAAAAAAGTACCTGTACCAGTGCCGGCCAAGAAACCAGATCGCACTATCGACGCTGATGTAGTTGTATTTGGGTCTGGGTTAGCAGGTCTTTCCGCGGCTGTGGAGGCCGCTGATTGTGGTGCGAAAGTCGTCCTGGTGGAGAAGCAAGGCATTGTGGGCGGGAGTAGTGCGATTAGTGGCGGCAAGCTTATCGCAGCGGACACACGTATGCAACGTGAACAAGGGATTTATGACTCTCCACAAGAGTTGTTTGGTTTCCTTAAGAATGCGGCCGGGGGATTCTTAGATGATCCCAAGATTAACTACTTTTGTTATCACGCGAATGAGAATTTAGAATGGCTTATTAAGATGGGGCATGAGGTGCAAGATTTAGAGGCTCCGCATGGTTCACAATTACCCTGGCGCATCCATAATTGTATCGGAGGTGAAGGCCAAACCATGGGATGGGGAGGTTCATTCATTGTGCCTTTGAATAATCGCTTCCACGAATTAGGAGGGACGACTTTGCTGAACACCGCGCTTAGCGAGCTTATTCGTGAGGATGGTCGTGTCGTAGGTGCTAAGGCCGTAGATACGCAAGATGGGAGCACCGTCACGTTCCACGCTAGCCAGGGTGTGATTCTTGGGACTGGAGGCTATGCGGCGAACCGTGAGTTAGTGGAAAGTAAATTCCCATGGATGAAGGACTACTACTATAATTGCCCGGATTCATCGCAGGGGGATGGCATCTGGGCGGCGGAAGCTATTGGAGCGCGCAACTATCAACATCCATATTTGCAGACAATGCTTCTGCACGACCGCTCCGGCGCAGGGGTCAACGAGGAAAGCGGGTTAATCGTTACTCGTTCAGGTAAGCGCTTCTGTAACGAGTACCAGTTCCATTCCTTAGTTGGGGCGGAGCTTGCCCGTACTGGAAGTGCTGGTGCGTGGTATATTACTTGCGGAGATGAACCTTTTCAATTGTTGAACTATGCTTTGACCTTACCCGACACACCCAAAGCTGGATCGATCAAGGAGCTGGCCGGAAAAATGGGAGTTGACCCTGAAGTCCTGGAGAATACTGTGAACCGTTACAATGAGTTATGTCGTGCCGGGTTTGACGAGGACTTTGAAAAGCCAGCGGGCCAGATGCACGAGTTAAAAGGGCCCGTGTACTACGCCGTATTCCTGAAACCGGCAACATCTATCACATTTGGCGGCTTACAAATCGACATTACGGGTCGCGTTCTGGATCAGGAAGGACGTATCATCCCTGGACTGTTCGCGTCCGGAGAAGTTGCTAATACGGGTAACTTTGGTCATGGGGTTCCTGCGTGCGGTTATTCCTTAGGGCACGCTCTGTGCTTTGGCCGCATTGCCGCGCGCACGGCTTGTCAACGTGAAATGCTGTAA |

**Table S3.** Synthetic DNA sequence used in this study^a^

| **Name** | **Sequence (5’→3’)** |
| --- | --- |
| *baiJ*  from *L. scindens* VPI 12708  (1,722 bp) | ATGGCACATTACGTACCGGGTGCGTACGAAGGTATCGGACGTGGTTATCGTGGGAAATTGATCGTGAATGTGACAGTGACAGAAGAACGCATCGAAAAAATCCAAATTGTTAAACACAAAGAAGTCCGTGGTCTTGCATGGGACTTGCCGACATCTCCCATCGAAGTAATCCCTCCGCAAATTATTGAGTACCAGTCGTTAAATATTCCGCTTGTCAACGGGGCCGATCTGACGAGCGCCGCGATTTTAGACGCGGTAGCGGCAGCCCTGAAAGCTGCTGGGGCGACAGACGAAGACATCGAACAGTTGCGCCAGGCGCCAGGCCCTGAAGCTCCAGAACCAAAGGATGAAGTGCGCACTGTG  GACGTCGCTGTATTTGGGGCCGGAGCCGGAGGGTTGGCCGCGGCTATTGAGGCAAAAGAAGGGGGCGCGGATGTCATCCTGATTGAGAAGCAAGGGATTACTGGTGGTTCTACTGCGCGCTCGGGAGGGAAGCTTCTTGGGGCAGGAACAAAATGGCAGAAAGAACAAGGAATCTATGATACTAAAGAGATGTGCTATGATTACCTTATGGAGGTTGGTAATCGTCGCGGTGACTTTATGGACGCAAGTAAGAATCGCTATTTGGTTGAGCACCTTAACGAGACACTGGACTGGTTAGGGACGATGGGGTATCAGGTCCAAGACGTCGAGGCGATCCATGTCTCTCTTCAACCCTGGCGTGTCCACAATTCCATGGGAGGCGGAGGGCAAACTAACGGCCAGGGTGGAGAGATTACTACACCTCTTACCCATCACTACGTCGACAAGCTTGGAGGGGAAATCTTATACAATACAGCATTGAAGGAATTGCTTACGGATGAGAACGGCACGGTGAACGGTGCGGTTTGTGAGAAATTAGACGGCTCGAAGCTGACCGTATACGCCAAAAAGGGGGTGATCCTTGCCACCGGCGGCTACAGTCGCAACAAGGAGATGTGCGCCCGCTATCCAGTCGCCCATTACTTTTCAACGACTCCCAAGAGCAACGTAGGAGAAGGCTTAATTGCAGCGGAAAAGATTGGGGCCCGTAATTTTGTGCACCCTGGAATTCAGGTAGTCTACACTAGTCTTACATGTGGTATTGGTATCAATGATGAATCTGGGCTTATCGTAAACGAACGTGGAGAACGTGTCGTGAACGAATGGAGTTATCAATACCACGTTTCTGACGCTTTAGCTGCCTCGGGTTCTAACTGTGGGTGGTACATCACCTCGGGCGACGAGCCCTATTCTGGTGTACAGTATGGCTTCAAACAAGCCGTCGAAGGCACCTCTCGTGATAAGGCAGCGGACAGTATCGAAGAATTGGCAGCCATGATTAAGTGTGACCCTGCGGTTTTGCGTGCGACCTTCGATCGCTATTCGGAATTAGTTGATAAGGGGGTGGATGAGGACTTTGGTAAGCCGTCTCGCTTCTTGCACCCAATCAATGGACCCAAATATGCTGCATTACGTTTACATCCATGCGTAACCGTGACATTTGGAGGATTGGAGACAGACGTGGCGGCCCGCGTACTGGACACTGAGGGTCGCCCAATCCCGGGTCTTTATGCTGCTGGTGAAGTAGCCGATACTGGCATGTTTGGCACAGAATACCCCACCTGCGGCACTTCGATCGGAGGAGCACTTTTCTACGGTCGTATCGCTGGACGCGTCGCTAGTGGGCAGAGTATGCTTTAA |

**Table S3.** Synthetic DNA sequence used in this study^a^

| **Name** | **Sequence (5’→3’)** |
| --- | --- |
| *“baiO”*  from *L. scindens* ATCC 35704  (1,659 bp) | ATGATCAGCATGATTCGTATCAATCAGCTGAAGTTAAACATCAAGCATTCTGAAGCTGACCTTAAGGAGAAAATTTTAAAAACTTTATGCATCTCGGAGGACAGTCTTTTGTCATATGAAATTAAGAAACAAAGTTTAGATGCACGTCGCAAACCAGAGCTTTACTATGTGTATGCTGTGGATGTGAAAGTAAAGAACGTATCTTCGATCAAGAAACGCGTCCGTAATCAGAATGTTCAATTTAAGGACAAACCGCTTTCTTACCAAATTCAAGCAAATGGCACAGAAGTCTTGCATCATCGTCCAGTTGTCATCGGGACTGGGCCTGCTGGCCTGTTCTGCGGGTACCAGCTGGCTGTCTTGGGTTACCGTCCGATCCTTCTTGAGCGTGGTGCATGTGTTGAAGAGCGCATGCAGGCGGTTGAGCGCTTCTGGGCAACCGGTGAACTGGACCAGAATTGTAACGTGCAATTCGGTGAGGGCGGGGCCGGAACTTTTTCGGATGGGAAATTAAATACATTGGTGCATGATAGCAATGGTCGTTCACAAAAAGTCCTTGAATTATTCGTGAAGTACGGGGCACCGAAGGAAATCTTATACCAGCACAAGCCCCACATCGGAACCGATGTTCTGAGTCGTGTGGTAAAGAACATCCGTGAGGCCATCTTATCCTATGGAGGCGAAGTCCGTTTCTTAACACGTGTAACCGACATCTTGTCTGAAAGCGCGGGTGAGGGTCGTCGTTTGACGGCCTTGCAAGTCTACGACCATGCAGCAGGCAAGGAATACGCACTTGAGACTGAAATTGCTGTATTGGCGATTGGTCATTCTGCGCGTGACACTTTTTCTATGTTACTTAAGAACGAAATTCCCATGGAATCAAAGTCGTTCGCCGTTGGTGTACGTATCGAGCATCCACAGGCAATGATTGACGAATTCCAATATGGAATGAAAAACGATGGGAGTCTGCCCCCGGCTAGTTACAAACTGACTGAAAATTTCGCGTGTGGACGCGGAGTCTATACCTTTTGTATGTGTCCGGGTGGGTATGTAGTGAATGCCTCATCGGAGCCCGGACGTCTGGCAGTTAATGGGATGTCATATCACGATCGTGATGGGTTCAATGCTAACAGTGCCGTTATCGTTACAGTGACGCCACAGGATTATGGGGGTACAGGAGTGTTAGCAGGGATGGAGTTCCAGCGTCGTTTAGAAGAGGCGGCGTACCGTCTTGGCAAAGGCCGTATCCCCGTGCAATTATTTGAAGATTTCTGCAAGAATCGTCCTTCCAAGGGTCCAGGAGATATCTTGCCTCAAATGAAAGGTGCATATGCGTGGAGTAATGTTCGTGAGATCTTCCCTCCCGAATTGTCCCGCGCCCTGGAAGAGGGAATCCGTTCATTCGACCGTAAAATTAAGGGGTACCCCCGCCCCGACGCTTTAGTGAGTGGAGTGGAATCTCGCACAAGCTCACCAGTGCGCATCTCCCGTGATGAGAGTATGCAATCTACGTTGTTCGGGTTGTACCCCTGTGGAGAAGGTGCGGGCTACGCTGGAGGTATCACCAGCGCAGCCATGGATGGTTTAAAAACCGCCGAAAGCATTGTCAAAAAATATCAATCACTGGACAAGCTGCCCAAAGGGCTGCGTAATTTCTAA |

**Table S3.** Synthetic DNA sequence used in this study^a^

| **Name** | **Sequence (5’→3’)** |
| --- | --- |
| *baiA1*  from *L. scindens* ATCC 35704  (774 bp) | ATGAGTATTATCGTCATCTCGGGATGCGCGACCGGGATCGGAGCGGCAACACGCAAGGTACTGGAAGCGGCTGGACACCAGATTGTTGGAATCGACATCCGTGACGCAGAGGTCATTGCTGATCTGAGTACGGCAGAAGGGCGCAAACAAGCAATCGCGGATGTTCTGGCAAAGTGTTCCAAGGGAATGGATGGCCTGGTACTTTGTGCCGGACTGGGTCCCCAAACAAAGGTCCTGGGTAATGTAGTGTCTGTGAACTACTTTGGTGCAACAGAATTGATGGATGCCTTCCTGCCTGCCTTGAAGAAAGGGCACCAGCCTGCCGCTGTTGTCATCTCAAGTGTGGCTAGTGCCCATCTTGCATTTGATAAAAATCCATTGGCGCTTGCCCTTGAAGCCGGGGAGGAGGCAAAAGCTCGTGCAATTGTCGAGCATGCGGGGGAACAGGGTGGGAATCTTGCCTACGCGGGTTCGAAAAACGCTCTGACGGTTGCAGTGCGTAAGCGTGCGGCCGCGTGGGGTGAAGCCGGAGTCCGCTTAAATACGATTGCCCCAGGTGCCACCGAGACTCCTTTACTGCAAGCTGGTCTTCAGGATCCCCGCTATGGTGAGAGCATCGCGAAGTTCGTCCCTCCCATGGGGCGCCGCGCGGAACCCAGCGAGATGGCAAGCGTAATCGCTTTCTTGATGTCACCAGCCGCGAGCTACGTGCATGGTGCTCAGATCGTGATTGATGGAGGCATTGATGCAGTTATGCGCCCGACCCAATTTTGA |

^a^ All *bai* genes were codon-optimized for *Escherichia coli* and synthesized.

**Table S4.** Summary calculations of number of gene hits in the MAG database, number of participants with the gene of interest, and disease information

| **Gene** | **Disease**  **State** | Number of Subjects w/at least 1 copy of the gene in their MAGs | Proportion of Subjects Normalized to Disease State | Total MAGs- gene present | Total MAGs – no gene/  absent | Total MAGs Proprotional to Disease State | Present MAGs/  Total MAGs | Present MAGs/  Absent MAGs | Median MAGs w/ gene in all MAGs in that disease state | Mean w/ gene in all MAGs in that disease state | Standard Deviation for all MAGs in that disease state | Median MAG's w/ gene in subset of participants w/ at least 1 copy of gene | Mean w/ gene in subset of participants w/ at least 1 copy of gene | Standard Deviation in subset of participants w/ at least 1 copy of gene |
| --- | --- | --- | --- | --- | --- | --- | --- | --- | --- | --- | --- | --- | --- | --- |
| ***baiP*** | **Healthy** | 52.000 | 0.196 | 53.000 | 6837.000 | 0.200 | 0.008 | 0.008 | 0.000 | 0.594 | 1.444 | 2.778 | 0.594 | 1.444 |
|  | **Adenoma** | 19.000 | 0.170 | 20.000 | 2414.000 | 0.179 | 0.008 | 0.008 | 0.000 | 0.946 | 1.909 | 3.030 | 3.611 | 2.071 |
|  | **Carcinoma** | 76.000 | 0.262 | 79.000 | 7533.000 | 0.272 | 0.010 | 0.010 | 0.000 | 0.800 | 2.525 | 3.175 | 4.060 | 4.403 |
| ***baiJ*** | **Healthy** | 59.000 | 0.223 | 59.000 | 6831.000 | 0.223 | 0.009 | 0.009 | 0.000 | 0.454 | 1.147 | 2.564 | 0.454 | 1.147 |
|  | **Adenoma** | 17.000 | 0.152 | 17.000 | 2417.000 | 0.152 | 0.007 | 0.007 | 0.000 | 0.947 | 1.854 | 2.941 | 3.476 | 1.958 |
|  | **Carcinoma** | 79.000 | 0.272 | 80.000 | 6831.000 | 0.276 | 0.012 | 0.012 | 0.000 | 0.938 | 2.783 | 3.226 | 4.196 | 4.606 |
| ***baiE*** | **Healthy** | 62.000 | 0.234 | 63.000 | 6827.000 | 0.238 | 0.009 | 0.009 | 0.000 | 0.640 | 1.469 | 2.778 | 0.640 | 1.469 |
|  | **Adenoma** | 21.000 | 0.188 | 21.000 | 2413.000 | 0.188 | 0.009 | 0.009 | 0.000 | 1.104 | 1.940 | 3.030 | 3.519 | 1.868 |
|  | **Carcinoma** | 91.000 | 0.314 | 94.000 | 7518.000 | 0.324 | 0.012 | 0.013 | 0.000 | 0.993 | 2.827 | 3.175 | 4.227 | 4.534 |
| ***baiCD*** | **Healthy** | 62.000 | 0.234 | 63.000 | 6827.000 | 0.238 | 0.009 | 0.009 | 0.000 | 0.562 | 1.379 | 2.703 | 0.562 | 1.379 |
|  | **Adenoma** | 19.000 | 0.170 | 19.000 | 2415.000 | 0.170 | 0.008 | 0.008 | 0.000 | 1.103 | 1.959 | 3.078 | 3.555 | 1.908 |
|  | **Carcinoma** | 90.000 | 0.310 | 94.000 | 7518.000 | 0.324 | 0.012 | 0.013 | 0.000 | 0.966 | 2.642 | 3.226 | 4.114 | 4.115 |

**Table S5.** Results from chi squared tests for associations of gene presence with participant disease status

|  | Chi Squared – association of gene presence/absence with disease state | | | |
| --- | --- | --- | --- | --- |
| Gene | X^2 | df | P-value^a^ | Directionality |
| *baiP* | 5.5112 | 2 | 0.06357 | Presence proportion higher toward carcinoma |
| *baiJ* | 6.8247 | 2 | 0.03296 | Presence proportion higher toward carcinoma |
| *baiE* | 8.3345 | 2 | 0.01549 | Presence proportion higher toward carcinoma |
| *baiCD* | 9.5482 | 2 | 0.00845 | Presence proportion higher toward carcinoma |

^a^ P-values less than 0.05 are designated as significant. Cells highlighted in **green** have a p-values < 0.05

**Fig. S1.**


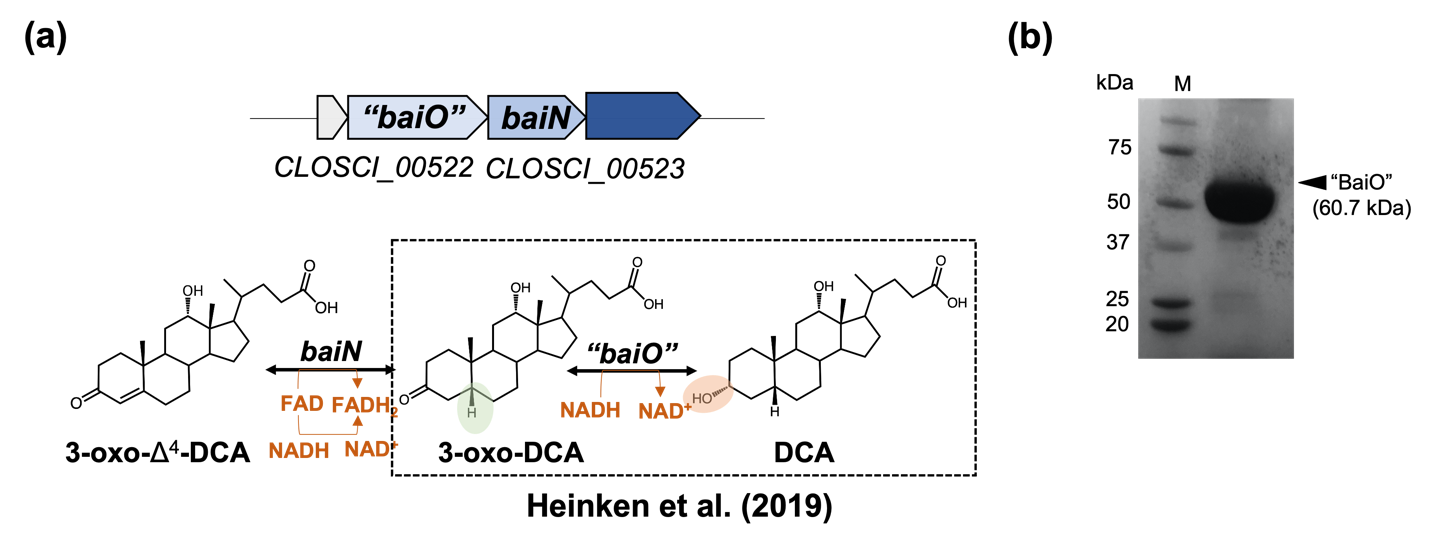


**Fig. S1. The “*baiO*” gene^1^ from *L. scindens* ATCC 35704 does not encode neither a bile acid 3ɑ-HSDH nor 3-oxo-**Δ^4^**-reductase.** (a) Gene organization of “*baiO*” with genomic context and expected formation of bile acid stereoisomers after reduction of 3-oxo-DCA by the “BaiO” enzymatic activity. (b) Heterologous expression of N-terminal his-tagged recombinant “BaiO” in *E. coli* BL21(DE3). SDS-PAGE confirms expression of 60.7 kDa recombinant “BaiO”.

**
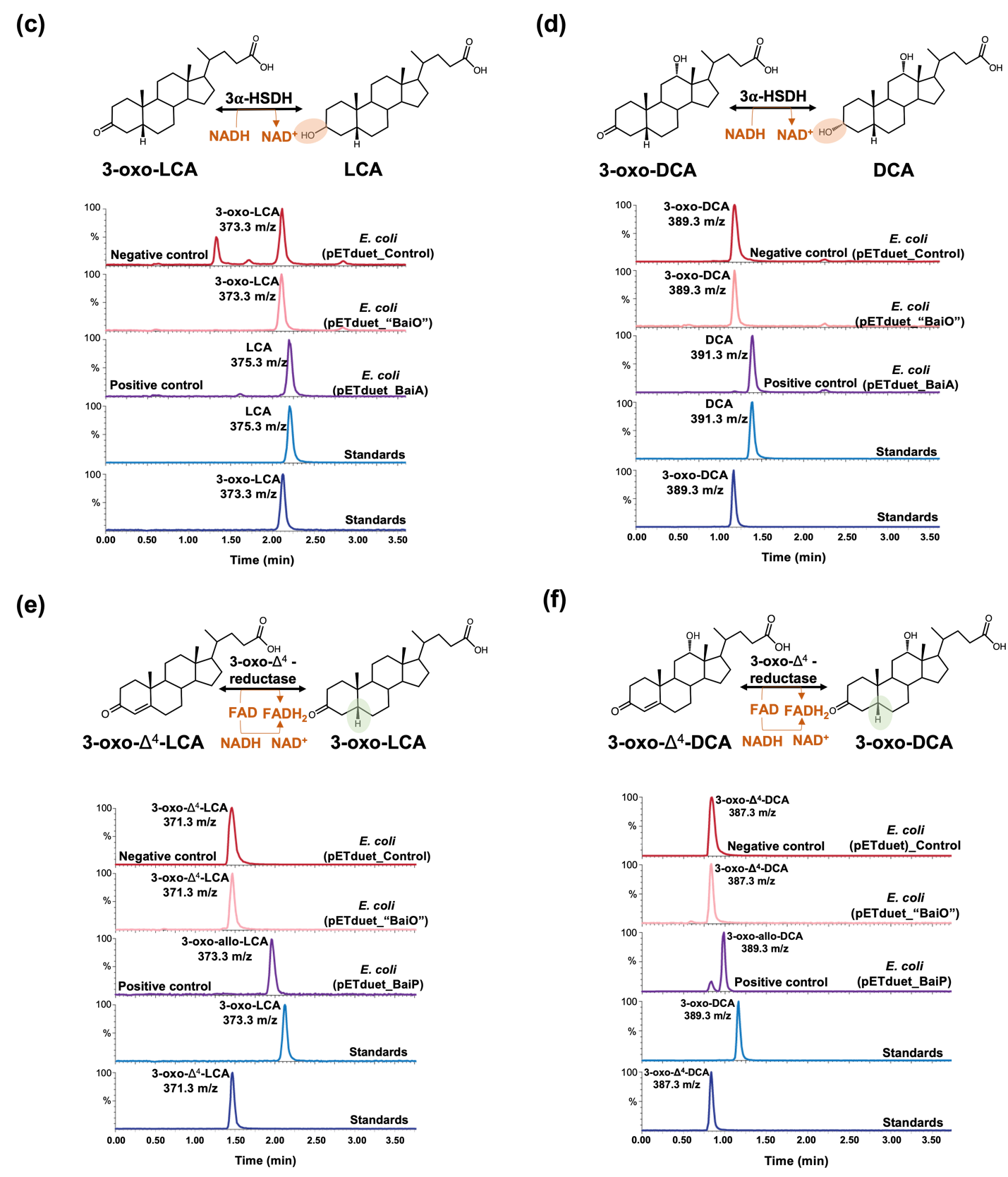
**

**Fig. S1. The “*baiO*” gene^1^ from *L. scindens* ATCC 35704 does not encode neither a bile acid bile acid 3ɑ-HSDH nor 3-oxo-**Δ^4^**-reductase.** Analysis of bile acid products after 24 h incubation of the *E. coli* expressing “*baiO*” in a resting cell assay with 50 uM (C) 3-oxo-LCA (d) 3-oxo-DCA, (e) 3-oxo-Δ^4^-LCA, or (f) 3-oxo-Δ^4^-DCA, did not yield a detectable product by LC/MS.

**
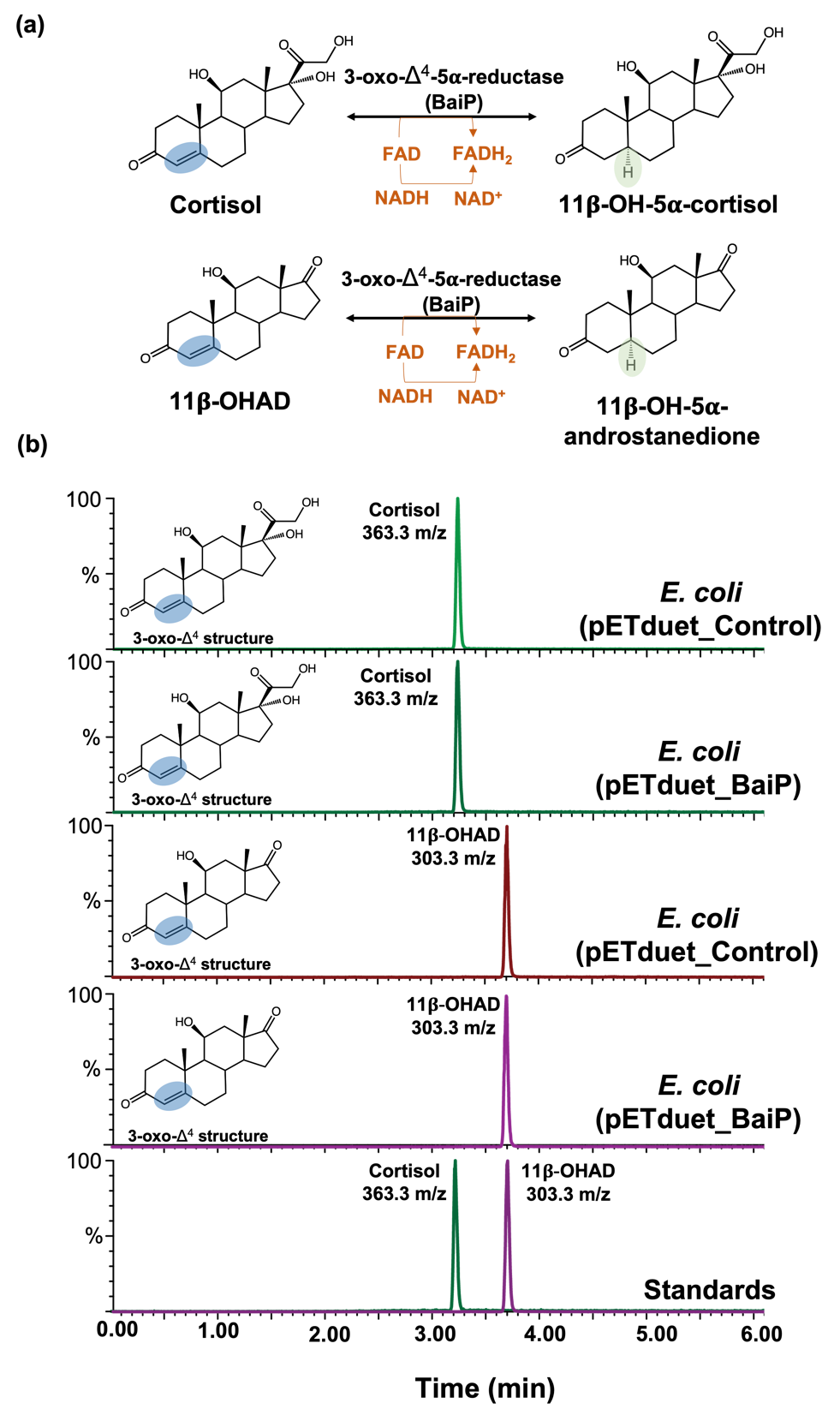
**

**Fig. S2. Cortisol and 11β-OHAD are not substrates for BaiP.** (a) Expected formation of steroid stereoisomers after reduction of cortisol and 11β-OHAD by the BaiP enzyme. (b) Representative LC/MS chromatographs after resting cell assay with *E. coli* BL21(DE3) pETduet_Control or pETduet_BaiP incubated in anaerobic PBS containing 50 μM Cortisol (Top panels 1 & 2) or 50 μM 11β-OHAD (Bottom panels 3 & 4). Standards are shown in Panel 5 (bottom).

**
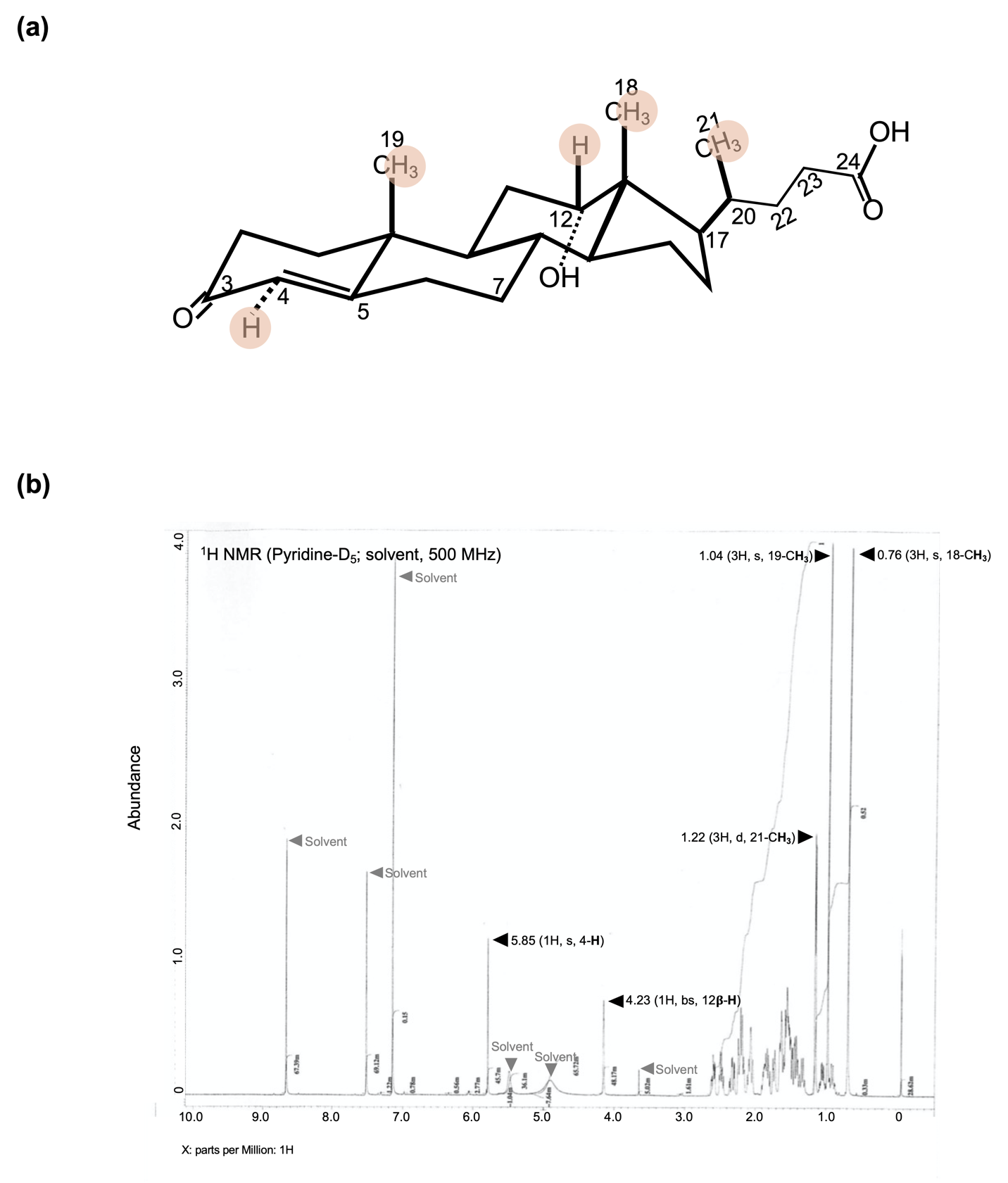
**

**Fig. S3. The molecular structure of the chemically synthesized 3-oxo-Δ^4^-DCA.** (a) Stereochemical formular of 3-oxo-Δ^4^-DCA (b) ^1^H-NMR spectra of the chemically synthesized 3-oxo- Δ^4^-DCA. ^1^H-NMR (pyridine-d_5_): *d :*0.76 (3H, s, 18-C**H_3_**), 1.04(3H, s, 19-C**H_3_**), 1.22 (3H, d, *J*=5.2 Hz, 21-C**H_3_**), 4.23 (1H, bs, 12β-**H** ), 5.85 (1H, s, 4-**H**). Solvent (pyridine-d_5_): *d*: 3.6, 4.9, 5.4, 7.1, 7.5, 8.7.


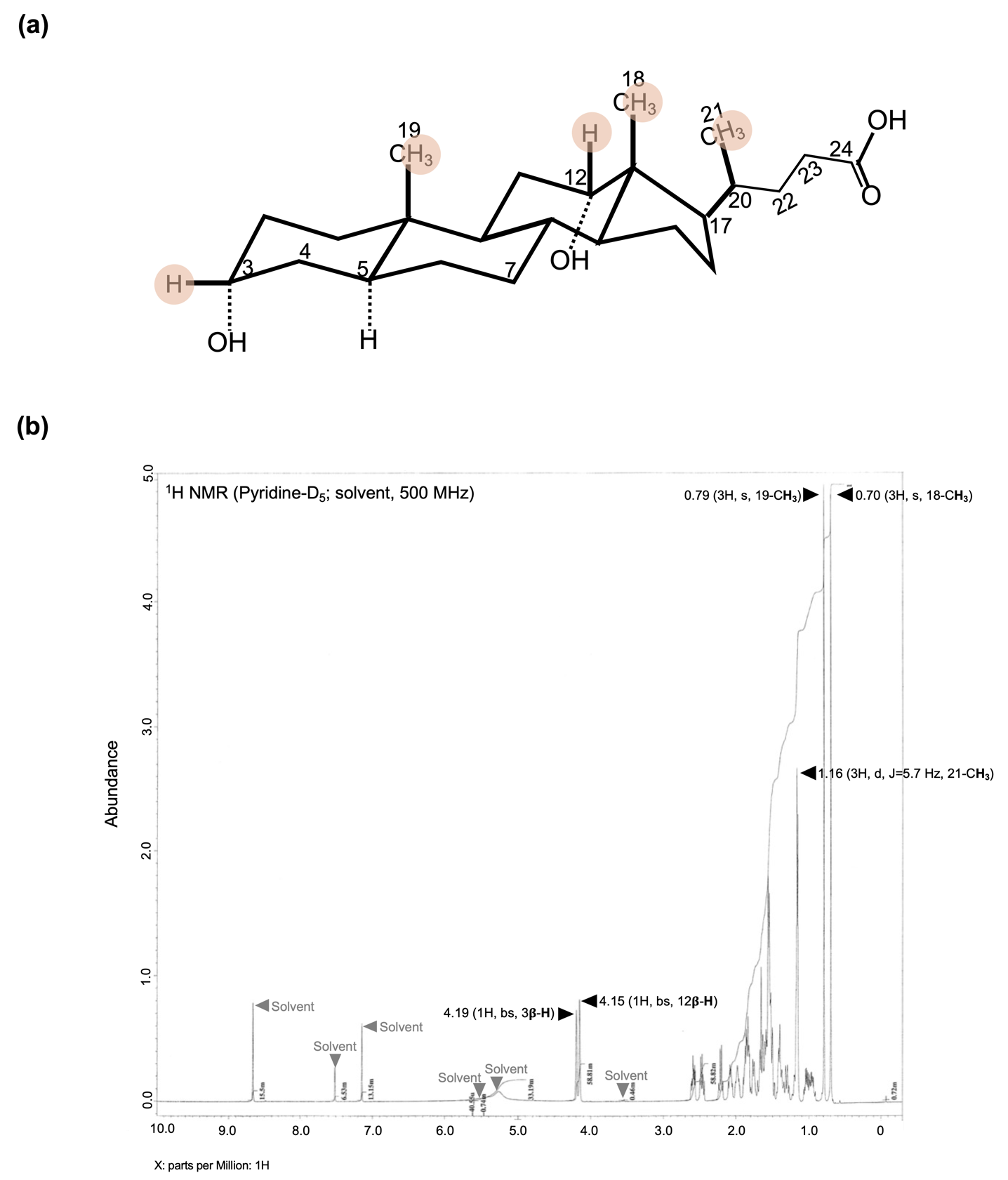


**Fig. S4. The molecular structure of the chemically synthesized allo-DCA.** (a) Stereochemical formular of allo-DCA (b) ^1^H-NMR spectra of the chemically synthesized allo-DCA. ^1^H-NMR (pyridine-d_5_): *d :*0.70 (3H, s, 18-C**H_3_**), 0.79 (3H, s, 19-C**H_3_**), 1.16 (3H, d, *J*=5.7 Hz, 21-C**H_3_**), 4.15 (1H, bs, 12**β-H** ), 4.19 (1H, bs, 3**β-H**). Solvent (pyridine-d_5_): *d*: 3.6, 5.3, 5.6, 7.1, 7.5, 8.7.

**
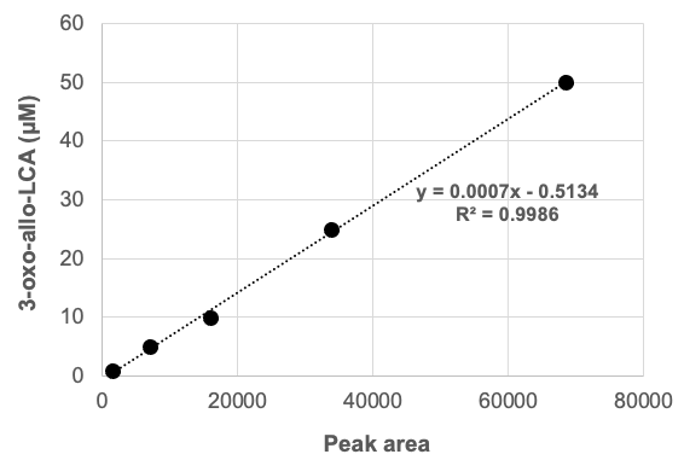
**

**Fig. S5.** Standard curve for calculating 3-oxo-allo-LCA concentration. The concentration was determined by the peak areas obtained from LC-MS analysis.

**References**

[1] Heinken A, Ravcheev DA, Baldini F, Heirendt L, Fleming RMT, Thiele I. Systematic assessment of secondary bile acid metabolism in gut microbes reveals distinct metabolic capabilities in inflammatory bowel disease. Microbiome 2019; 7:75.
